# Exercise and apulian hypocaloric diet affect adipokine changes and gastric banding-induced weight loss: A prospective study on severe obese subjects

**DOI:** 10.1016/j.amsu.2020.02.005

**Published:** 2020-02-25

**Authors:** Gabriella Garruti, Michele De Fazio, Palma Capuano, Gennaro Martinez, Maria T. Rotelli, Francesco Puglisi, Nicola Palasciano, Francesco Giorgino

**Affiliations:** a Department of Emergency and Organ Transplantation, Section of Internal Medicine, Endocrinology, Andrology and Metabolic Diseases, University of Bari Aldo Moro, Piazza G. Cesare 11, 70124, Bari, Italy; b Department of Emergency and Organ Transplantation, Section of General Surgery and Liver Transplantation, University of Bari Aldo Moro, Piazza G. Cesare 11, 70124, Bari, Italy

**Keywords:** Apulian diet, Bariatric surgery, Lifestyle, Obesity, Physical activity, Resistin

## Abstract

**Background:**

Adiponectin and Resistin correlate with insulin sensitivity and cardiovascular risk, respectively. This study aimed to identify lifestyle factors that modulate changes in Adiponectin and Resistin levels after gastric banding positioning (LapGB).

**Materials and methods:**

Before (T0), 3 months (T3), 6 months (T6), and 12 months (T12) after LapGB, serum Adiponectin and Resistin levels were evaluated in a single-centre prospective study including a cohort of 27 non-diabetic obese subjects (S-Ob, BMI ≥35 kg/m^2^). After surgery, a dietitian checked the adherence of S-Ob to an Apulian hypocaloric diet (aphypoD)/physical activity (phA) and, according to their high or low compliance to aphypoD/phA, S-Ob were included in group 1 (n = 14) or 2 (n = 13) respectively. Serum Adiponectin and Resistin were also measured in 10 healthy controls.

**Results:**

At baseline, Resistin levels were significantly higher and Adiponectin levels significantly lower in S-Ob than in controls. After surgery, group 1 showed a 50.2% excess weight loss (%EWL), significantly decreased Resistin levels at T12 and increased Adiponectin levels at both T6 and T12 as compared with baseline. Group 2 showed 24.6 %EWL at T12, decreased Adiponectin levels at T6 and T12 as compared with baseline, but unaltered Resistin levels. After surgery, group 1 followed aphypoD/phA, while group 2 did not.

**Conclusions:**

LapGB fails to improve cardiovascular risk markers (Resistin) in S-Ob not improving lifestyle. Future studies might investigate these findings in a larger cohort and display whether aphypoD is more effective than other dietary intervention on cardiovascular risk in subjects undergoing LapGB or other Bariatric procedures.

## Highlights

• Gastric banding (LapGB) plus a 12-month lifestyle program was followed by a % excess weight loss >40.
• Resistin significantly declined in subjects adhering to a 1-year LapGB/lifestyle intervention.
• In subjects not following any lifestyle program after surgery, Adiponectin unexpectedly decreased.
• LapGB fails to change cardiovascular risk or insulin sensitivity without a healthy lifestyle.

## 1 Introduction

Adjustable gastric banding is a gastro-restrictive bariatric procedure attained by laparoscopic positioning of an adjustable silicone ring around the upper segment of the stomach. After closure of the ring, the stomach takes on the appearance of an asymmetrical egg-timer, with a small upper gastric pouch (calibrated at about 20 ml) and the remaining stomach below the band. This surgical technique is considered as “gastric restrictive” because it produces a significant reduction of the stomach volume and is associated with the feeling of early satiety [1,2].

Adiponectin and Resistin are cytokines playing a role in chronic low-grade inflammation. It is known that inflammation and endothelial dysfunction are involved in atherosclerotic plaque formation, and that dysregulation of adipokines in human obesity may contribute to this process [3,4]. Resistin is highly secreted during inflammation and endorses the production of pro-inflammatory citokines such as TNF-alpha, interleukin-6 and -12 [5]. It also plays a role in the upregulation of the expression of chemokines and adhesion molecules [6]. In humans, circulating levels of Resistin positively correlate with atherosclerotic cardiovascular disease [7], and some studies demonstrated that they are also increased in acute myocardial infarction [[8], [9], [10], [11]].

In this study we analyzed lifestyle factors that can modulate the changes in Resistin circulating levels and some surrogate markers of insulin sensitivity (HOMA-IR, Adiponectin) in a cohort of severely obese subjects (S-Ob) consecutively undergoing the positioning of laparoscopic adjustable gastric banding (LapGB). We found that, following bariatric surgery, subjects showing a good compliance to healthy lifestyle changes experienced a significant weight loss and reduction in Resistin.

## 2 Methodology

This was a prospective study including 27 patients (17 females, 10 males) consecutively followed at the Outpatient Obesity Clinic of the Unit of Endocrinology of the University Hospital “Consorziale Policlinico” in Bari, Italy. The inclusion criteria were age between 20 and 55 years and BMI 35–65 kg/m^2^. The exclusion criteria were current smoking, pregnancy, Type 2 and Type 1 diabetes mellitus, gestational diabetes, or other specific types of diabetes related to chronic pancreatitis, oral steroid therapy.

The study was conducted in compliance with the Declaration of Helsinki and European Guidelines on Good Clinical Practice. The Research Registry Unique Identifying Number for the study is Researchregistry5052.

Table 1 shows the baseline characteristics of the 27 subjects. After surgery, patients were divided in group 1 or 2 based on their "compliance" to a lifestyle intervention program including diet and physical activity. The lifestyle intervention consisted of nutritional interviews promoting a mildly hypocaloric Apulian diet (aphypoD) plus daily physical activity. Four times a year, the S-Ob were regularly interviewed by a dietitian to check whether they followed a mildly hypocaloric diet (daily caloric deficit of 500 Kcal/day) and performed physical activity. Subjects with high compliance to lifestyle intervention were included in group 1 and subjects with low compliance to lifestyle intervention in group 2. We defined the above mentioned regimen as mildly hypocaloric Apulian diet (aphypoD) because it consisted of Mediterranean-like meals [13], including specific food items typically present in the diet from the Apulia region in Italy such as wholegrain “friselle” with raw tomatoes, wholegrain “orecchiette”, turnip tops (Italian equivalent “cime di rape”), raw olives and olive oill used as dressing [14]. Blood samples were taken pre-operatively (T0), and 3 months (T3), 6 months (T6) and 12 months (T12) after surgery, from the antecubital vein for measurements of glucose, insulin, total cholesterol (Chol), high-density lipoprotein (HDL) Chol, low-density lipoprotein (LDL)-Chol, triglycerides, and liver function tests (alanine amminotransferase, ALT, aspartate aminotransferase, AST, γ glutamyl transpeptidase, γGT). Serum Adiponectin levels were measured by B-Bridge human adiponectin ELISA Kit (B-Bridge International, Inc., Otsuka Pharmaceuticals, Japan), and serum Resistin levels by Mediagnost Enzyme Immunoassay for Quantitative Determination of human resistin (Mediagnostic, Aspenhaustr. 25 • D-72770 Reutlingen, Germany) (see Table 2).

**Table 1:** Description and comparison of basal variables in 27 morbidly obese patients and in those of Group 1 and 2.

Variable	Total (n = 27) Mean/SEM Median	Group 1 (n = 14) Mean/SEM Median	Group 2 (n = 13) Mean/SEM Median	p-value
Age (years)	35.42/3.04 32.0	34.42/3.55 34.5	37.14/6.46 27.0	0.67
Duration of Obesity (years)	22.23/4.04 20.0	17.63/4.095 15.0	29.6/7.65 23.0	0.16
BMI (Kg/m2)	47.58/1.45 48.27	48.61/6.14 47.86	45.53/2.33 48.73	0.40
Waist (ATP III) (cm)	138.61/3.24 143.0	140.83/12.26 139.5	134.78/6.23 143.0	0.36
Hip (cm)	136.61/3.182 138.0	140.42/13.57 140.0	130.07/4.29 135.0	0.11
Waist/Hip ratio	1.019/0.013 1.021	1.007/0.338 1.023	1.036/0.029 1.018	0.55
Neck (cm)	42.88/1.16 41.25	44.95/4.41 46.0	40.5/1.38 40.0	***0.05***
SPB (mmHg)	129.57/4.38 128.0	134.25/17.77 135.0	123.33/4.98 126.5	0.23
DBP (mmHg)	82.429/2.85 85.0	84.5/12.44 90.0	79.66/3.0 82.0	0.42
Heart rate (b/min)	78.0/2.75 76.0	82.0/12.44 78.0	75.33/1.77 74.0	0.26
HOMA-IR	6.53/1.79 4.70	8.03/2.672 4.485	3.91/0.97 4.435	0.29
Matsuda index	2.524/0.28 2.754	2.71/0.45 2.767	2.27/0.31 2.155	0.49
Fasting Glucose (mg/dl)	93.89/3.26 96,2	96.82/3.30 102	88.6/6.81 85	0.30
Fasting Insulin (mIU/l)	26.85/6.51 20.5	32.31/9.62 20.5	17.37/3.98 18.90	0.29

Comparisons between variables of Group 1 and Group 2 were performed with Mann Whitney *U* Test; p values are referred to comparison between Group1 and Group 2.

BMI: body mass index; DBP: diastolic arterial blood pressure; HOMA: Homeostasis model assessment; IR: Insulin resistance; SBP: systolic arterial blood pressure; SEM: Standard error mean.

**Table 2:** Description and comparison of liver function tests and lipid pattern in 27 morbidly obese subjects and in those of Group 1 and 2.

Variable	Total (n = 27) Mean/SEM Median	Group 1 (n = 14) Mean/SEM Median	Group 2 (n = 13) Mean/SEM Median	p-value
AST (U/l)	61.0/21.55 33	75.2/33.34 33.0	37.33/13.76 33.0	0.44
ALT (U/l)	52.63/16.06 41.5	63.4/26.02 48.0	34.67/6.99 39.0	0.43
γGT(U/l)	43.71/9.72 35.0	44/13.17 35.0	43/16.1 43.0	0.97
HDL Cholesterol (mg/dl)	51.2/3.55 49.2	53.23/4.5 51.20	49.17/5.77 44.5	0.59
LDL Cholesterol (mg/dl)	128.92/9.84 121.0	126.42/14.09 121.0	131/15.84 126	0.84
Total Cholesterol (mg/dl)	199.86/12.93 193.0	200.37/15.86 201.5	199.17/23.31 183.0	0.97
TGL (mg/dl)	131.29/16.86 108.5	153.13/22.2 132.0	102.17/22.33 95.5	0.14

Comparisons between variables of Group 1 and Group 2 were performed with Mann Whitney *U* Test; p values are referred to comparisons between Group1 and Group 2.

ALT: Alanine Aminotransferase; AST: Aspartate Aminotransferase; γGT: γ Glutamil Transpeptidase, HDL: High-density lipoprotein ; LDL: Low-density lipoprotein ; TGL: Triglycerides.

Before and after surgery, physical activity levels were recorded using the short form of International Physical Activity Questionnaire (IPAQ). IPAQ is a good instrument to facilitate surveillance of physical activity based on a global standard [[15], [16], [17]]. The short form includes the activity of four intensity levels: 1) vigorous-intensity activity such as aerobics, 2) moderate-intensity activity such as leisure cycling, 3) walking, and 4) sitting. Before and following bariatric surgery, the nutritional evaluation (average daily energy and nutrient intake) was carried out by trained dietitians and recorded according with the Italian Food composition database. In group 1, the dietary compliance was assessed every month, in the first 3 months after surgery, and then after 6 and 12 months to obtain information about adherence to aphypoD. Subjects of group 2 did not undergo any lifestyle change. Subjects from both groups were monitored before and 3, 6 and 12 months after surgery by an endocrinologist to prescribe vitamins and electrolyte support. The work has been reported in line with the STROCSS criteria [18].

## 3 Statistical analysis

NCSS Statistical software (NCSS LLC | 329 N 1000 E, Kaysville, UT 84037) was used for statistical analysis. For normally distributed data, the results are presented as mean ± standard deviation (SD) unless stated otherwise. Comparisons between independent groups were performed with the Student's *t*-Test for normally distributed data. For non-normally distributed data, Wilcoxon signed-ranks test and Mann–Whitney *U* test were used and p-values <0.05 were considered statistically significant. Repeated Measures Anova tested comparisons between variables in different groups at different time-points.

The sample size was calculated assuming a 35% difference in response between the two groups (high/low compliance). We estimated that 20 patients would have been the number required for the study to have 80% power and an α error of 5%. A per-protocol analysis was applied to the trial. Instead of 20 subjects we recruited 27 subjects (7 more subjects) because we were expected to have some dropouts. The randomization list was generated using the online resource available at www.randomization.com.

## 4 Results

The mean age of subjects was 35.42 years (SEM 3.04; median 32 years), with a mean body mass index (BMI) of 47.58 kg/m^2^ (SEM 1.45; median 48.27) (range 36.77–57.86). Comorbidities included arterial hypertension (9 patients, 4 in group 1, 5 in group 2), joint disease (4 patients, 2 in group 1, 2 in group 2), dyslipidemia (8 patients, 3 in group 1, 5 in group 2), gallbladder stones (1 patient in group 1), anxiety-depression (3 patients in group 1), cardio-respiratory diseases (Obstructive Sleep Apnea Syndrome in 3 patients, 1 in group 1, 2 in group 2), and vascular disease (5 patients, 2 in group 1, 3 in group 2). Baseline anthropometric variables, arterial blood pressure levels, heart rate, lipid pattern, liver function tests, HOMA-IR and Matsuda index [19] are shown in Table 1.

Before surgery, S-Ob were all deemed sedentary since their leisure time activity was 300 kcal/day (1255 kJ/d).

At T0, Resistin levels were significantly higher and adiponectin levels significantly lower in S-Ob than in controls (p = 0.005343 and p = 0.000035, respectively). In all S-Ob, Adiponectin levels were not significantly different at T6 and T12 compared with T0 (p = 0.9287, p = 0.9185), while Resistin levels showed a non-significant decrease at T6 (p = 0.0948) and a significant decrease at T12 as compared with T0 (p = 0.0211).

**Fig. 1:**
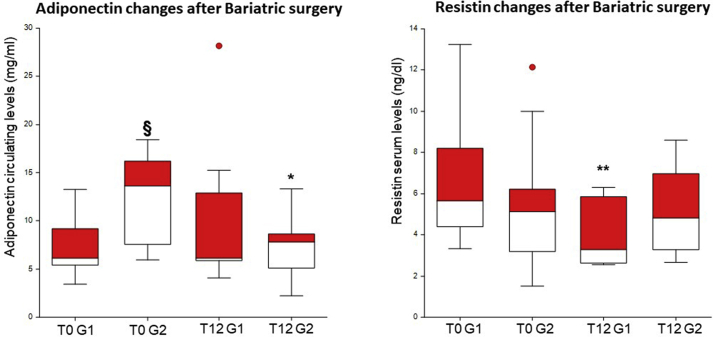
(A) Box plot of circulating Adiponectin levels in S-Ob of Group 1 (G1) (n = 14) and Group 2 (G2) (n = 13) before (T0) and 12 months (T12) after bariatric surgery. Comparisons between circulating Adiponectin levels at T0 and T12 were performed with T-test within the same group. Comparisons between groups were performed with Mann Whitney *U* test. At T0 Adiponectin levels of G1 were significantly different from those of G2 (§) p = 0.022. In G2 Adiponectin levels at T12 were significantly different from those at T0, (*) p = 0.05. Repeated Measures Anova tested comparisons between variables in different groups at different time-points and did not confirm any significant change in Adiponectin. Error bars indicate SD. (B) Boxplot of circulating Resistin levels in S-Ob of Group 1 (G1) (n = 14) and Group 2 (G 2) (n = 13) before (T0) and 12 (T12) months after bariatric surgery. Comparisons between circulating Resistin levels at T0 and T12 were performed with T-test within the same group. Comparisons between groups were performed with Mann Whitney *U* test; Resistin levels at T12 were significantly different from those at T0 in SObs of G1, (**) p = 0.004. Group 1 subjects showed a significant decrease in Resistin levels at T12 (p = 0.004) Repeated Measures Anova tested comparisons between variables in different groups at different time-points. This analysis confirmed the significant decrease in Resistin levels at T12 in Group 1 only (Repeated Measures ANOVA: F-ratio = 3.17, p = 0.037). Error bars indicate SD.

Following bariatric surgery, patients were stratified according to their compliance to both aphypoD and physical activity [[15], [16], [17],^20^]. After surgery, 14 S-Ob (Group 1) achieved a mean %EWL of 50.2%, showed a good compliance to aphypoD and declared on a questionnaire to walk at least 30 min/day (IPAQ score: 3). Group 1 subjects showed a significant decrease in Resistin levels at T12 (p = 0.004) (Repeated Measures ANOVA: F-ratio = 3.17, p = 0.037), a significant increase in Adiponectin levels at T6 (p = 0.0018), and a non-significant increase in Adiponectin levels at T12, as compared with T0 (Table 3, Table 4; Fig. 1) (see Table 5).

**Table 3:** Adiponectin and Resistin levels before and 3, 6, 12 months after surgery in 27 morbidly obese subjects.

**Adiponectin**				
Time points	**T0**	**T3**	**T6**	**T12**
Mean (mg/ml)	11.06	11.25	10.72	8.82
SEM	1.73	1.50	1.49	1.44
Median(mg/ml)	7.57	8.86	8.43	7.72
p		0.93	0.93	0.92
**Resistin**				
Time points	**T0**	**T3**	**T6**	**T12**
Mean (ng/ml)	6.05	5.14	4.92	4.50
SEM	0.58	0.38	0.44	0.45
Median (ng/ml)	5.19	4.37	4.53	3.63
p		0.10	0.10	**0.02**

In 9 controls Adiponectin mean circulating levels were 26.76 mg/ml (SEM:2.26 Median:25.08) and Resistin mean circulating levels were 2.891 ng/ml (SEM:0.53; Median:2.541). Comparisons between each time point and T0 were performed with T-test.

SEM: standard error mean; p values are referred to differences between each time point and T0.

**Table 4:** Adiponectin levels before and 3, 6, 12 months after surgery in subjects of Group 1 and 2.

Time points	(T0)	(T3)	(T6)	T12
**Group 1**				
Mean (mg/ml)	7.23	7.75	10.01	10.12
SEM	0.88	0.94	1.28	2.73
Median (mg/ml)	6.12	6.74	9.46	6.11
p		0.08	**0.002**	0.10
**Group 2**				
Mean (mg/ml)	14.17	13.66	11.22	7.35
SEM	3.26	2.67	3.37	1.14
Median (mg/ml)	13.60	14.26	7.70	7.81
p		0.79	**0.05**	**0.05**

Comparisons between each time point and T0 were performed with T-test within the same group; p values are referred to each time point as compared with T0; SEM: standard error mean; p values are referred to differences between each time point and T0. Repeated Measures ANOVA did not confirm any significant change in Adiponectin.

**Table 5:** Resistin levels before and 3, 6, 12 months after surgery in subjects of Group 1 and 2.

Time points	(T0)	(T3)	(T6)	T12
**Group 1**				
Mean (ng/ml)	6.51	6.09	7.02	3.98
SEM	0.81	0.65	1.18	0.42
Median (ng/ml)	5.66	5.36	4.89	3.27
p (versus T0)		0.57	0.65	**0.004**
**Group 2**				
Mean (ng/ml)	5.41	4.71	4.85	5.03
SEM	0.90	0.77	1.03	0.70
Median (ng/ml)	5.12	3.47	3.36	4.82
p (versus T0)		0.64	0.73	0.97

Comparisons between each time point and T0 were performed with T-Test within the same group; p values are referred to each time point as compared with T0; SEM: standard error mean. Repeated Measures Anova tested comparisons between variables in different groups at different time-points.

Group 1 subjects showed a significant decrease in Resistin levels at T12 (p = 0.004) (Repeated Measures ANOVA: F-ratio = 3.17, p = 0.037).

Thirteen subjects (Group 2) did not specifically follow any program of lifestyle changes and remained sedentary (IPAQ score: 4). As compared with T0, Group 2 subjects achieved a mean %EWL of 24.6% at T12 and showed a significant decrease in Adiponectin levels at T6 (p = 0.0493) and T12 (p = 0.05), and unchanged Resistin levels.

We retrospectively analyzed pre-surgery HOMA-IR and Matsuda index in Group 1 and Group 2 subjects. Both baseline HOMA-IR and Matsuda index were not significantly different in subjects belonging to the two groups and overall (Table 1). All anthropometric indices (BMI, waist, hip, neck circumference) were not different in Group 1 and Group 2 subjects except for neck circumference, which was significantly higher in Group 1 subjects (p = 0.0448). Baseline circulating levels of glucose, insulin, total Chol, HDL Chol, LDL-Chol, triglycerides, ALT, AST, γGT were also not significantly different between the two groups.

## 5 Discussion

In both genders, severe obesity is known to be associated with a mortality risk which is twice than that in the normal-weight population. Bariatric surgery produces a significant reduction in body weight and body fat mass in operated patients, improves all comorbidities associated with obesity, as well as the quality of life, and reduces the mortality rate [12,[21], [22], [23], [24], [25], [26], [27], [28]]. Nonetheless, among different bariatric procedures, adjustable gastric banding does not modify the topographic anatomy of the stomach nor the absorption of nutrients and works by reducing the amount of food which is ingested during each meal. Therefore, it represents a slightly different context to assess the effects of post-bariatric surgery lifestyle changes as compared with other bariatric procedures.

Following surgery, Adiponectin levels were not significantly different from values at baseline, while Resistin levels showed a significant decrease at T12 as compared with values at T0. Based on these data we could argue that bariatric surgery was generally associated with an improvement in the chronic low-grade inflammation.

Patients operated by LapGB were stratified according to their compliance to aphypoD and according to the short form of IPAQ too [[15], [16], [17]]. Fourteen subjects changed their lifestyle according to nutritionists’ advices (Group 1), declaring to perform at least a 30-min walk every day (score 3 according with IPAQ) after surgery. Thirteen subjects showed a poor compliance to activate lifestyle changes (Group 2) and remained sedentary (score 4 according with IPAQ). Group 1 subjects reduced their energy intake of 500 kcal/day or more and showed to be adapted to Apulian dietary habits. By contrast, Group 2 subjects reduced energy intake but still assumed sweet beverages and cakes often during the week and only rarely ate vegetables, legumes and fish. Six and 12 months after surgery, Resistin levels were not significantly different in Group 2 either from T0 or from controls. Thus, despite LapGB-induced weight loss, cardiovascular risk was not improving in these individuals. Moreover, Group 2 subjects showed an impairment in Adiponectin, which is a surrogate marker of insulin sensitivity, since circulating Adiponectin levels were significantly decreased 6 months and even more 12 months after surgery.

Interestingly, SObs of Group 1 not only achieved a higher mean %EWL as compared with those of Group 2, but also had a significant decrease in Resistin levels at T12 as compared with T0. In humans, few reports analyzed the effects of lifestyle changes on Resistin and Adiponectin circulating levels. In apolipoprotein-E knockout (ApoE^−/−^) mice with pre-existing atherosclerotic plaques, fed either a Western diet (WD) or a normal diet (ND) It was found that WD animals that did exercise had matrix metalloproteinase (MMP) activity close to that of ND animals, while ND animals that did or did not exercise had similarly low MMP activities. In the same study, Adiponectin and Resistin were negatively and positively correlated with atheromatous MMP activity, respectively [29]. These pre-clinical data demonstrate that diet quality and exercise may affect atheromatous MMP activity by modulating the chronic low-grade inflammation and relevant adipokines.

We analyzed pre-surgery HOMA-IR and Matsuda index in both groups. HOMA-IR is a surrogate marker of insulin resistance. Matsuda index represents both hepatic and peripheral tissue sensitivity to Insulin. In subjects with normal glucose tolerance, there is a good correlation between Matsuda index and hyper-insulinemic euglycemic clamp results (correlation coefficient 0.73, p < 0.0001) [19]. We found that both basal HOMA-IR and Matsuda index were not significantly different in subjects of different groups and overall (Table 1). Therefore, we can exclude that different basal levels of insulin sensitivity might have accounted for the different trends in post-surgery Adiponectin and Resistin circulating levels in the different groups. Basal circulating levels of glucose, insulin, total Chol, HDL Chol, LDL-Chol, triglycerides, ALT, AST, γGT were also not significantly different in SObs of Group 1 and Group 2. All anthropometric indices (BMI, waist, hip) were not different in SObs of different groups except for the neck circumference which was significantly higher in subjects of Group 1 as compared with those of Group 2. We might argue that subjects of Group 1 were less “metabolically healthy” [30] than those of Group 2, because neck circumference is associated with upper-body subcutaneous fat and non-alcoholic fatty liver disease (NAFLD) [[31], [32], [33]]. However, after surgery SObs of Group 1 improved their lifestyle more those of Group 2.

Few studies have analyzed circulating Resistin changes in obese subjects undergoing bariatric surgery. In a recent paper, Parreño Caparrós et al. found no significant differences in mean Resistin levels between morbidly obese patients and normal-weight controls [34]. These results are in contrast with our results. However, in the Spanish cohort from Parreño Caparrós et al. Resistin values of both normal-weight and obese subjects were higher than Resistin values found in other cohorts. In 2003, Considine et al. found 47% higher Resistin levels in serum from obese subjects compared with lean subjects. In this paper, the mean values for Resistin were 5.3 ng/ml (range 1.8–17.9) and 3.6 ng/ml (range 1.5–9.9) in in obese and normal-weight subjects, respectively [35]. Measured Resistin levels in our study cohort were in line with those reported by Considine's group [35].

In addition, Parreño Caparrós et al. did not find any change in circulating Resistin levels after gastric bypass surgery. The possibility exists that factors produced in the portion of the alimentary limb, which is preserved by the positioning of gastric banding, but is functionally excluded in gastric bypass [36,37], might have affected Resistin levels in our cohort. However, microarray data demonstrated that some genes, including Resistin, are differentially expressed in blood after bariatric surgery and a greater downregulation of Resistin expression was reported in obese diabetic subjects who lost more 10% excess body weight and in those who underwent gastric bypass as compared to gastric banding [38].

In few papers concerning subjects undergoing bariatric surgery, lifestyle changes are extensively analyzed. In the Longitudinal Assessment of Bariatric Surgery (LABS) study, Courcoulas et al. utilized growth mixture models to assess weight change trajectories for each participant undergoing either gastric bypass or laparoscopic adjustable gastric banding and to classify participants with similar modeled trajectories into groups. The Authors noticed that not all patients respond the same way to surgery, and they identified very different weight-loss trajectories, as well as varied improvements in hypertension, diabetes, and dyslipidemia and in rates of mortality and reoperation. They concluded that there is a substantial variability in response to treatment and suggest the opportunity to pay more attention to patient selection and education prior to surgery as well as enhance support for continued adherence to lifestyle changes in the postoperative years [39]. These results are in line with a previous report form our group, demonstrating hemorheological changes in obese subjects undergoing laparoscopic adjustable gastric banding plus lifestyle changes [12]. Noteworthy, also in the present study appropriate lifestyle changes could predict the reduction of pro-inflammatory/pro-atherosclerotic markers such as Resistin in S-Ob. The exercise-induced anti-inflammatory effect and the benificial changes in metabolome mediated by leisure-time physical activity have already been measured in some papers [[40], [41], [42], [43]]. In Type 2 obese diabetic patients exercise training reduces Resistin and other inflammatory markers (IL-6, IL18, CRP) [[40], [41], [42]]. Ther strength of our study is the detailed analysis of patient lifestyle after gastric restrictive surgery. The limitations of the study are the small number of subjects included in the study and the lack of comparison with other bariatric procedures.

In conclusion, despite S-Ob underwent the same bariatric procedure (gastric restrictive surgery), one year after surgery, S-Ob not changing their lifestyle display an impairment in Adiponectin, while those following a healthy lifestyle experienced a LapGB-induced %EWL >40% and a significant improvement in Resistin, a surrogate marker of inflammation and cardiovascular risk. From our data it seems that LapGB alone fails to improve cardiovascular risk markers (Resistin) or insulin sensitivity (Adiponectin) in obese subjects not improving their lifestyle. Future studies might assess whether Apulian diet and lifestyle are more effective than other dietary intervention on cardiovascular risk in severe obese subjects undergoing LapGB or other Bariatric procedures.

## Funds

The work was not supported or sponsored by additional funding agency of grant except for funds annually provided by the University of Bari “Aldo Moro”.

## Ethical approval

The study is a single-centre prospective study including a cohort of 27 nondiabetic obese subjects.

The study was conducted in compliance with the Declaration of Helsinki and European Guidelines on Good Clinical Practice. All subjects signed the informed consent before surgery and some of them were included in a previous paper from our group [ref.12, Capuano P, Catalano G, Garruti G et al. The effects of weight loss due to gastric banding and lifestyle modification on red blood cell aggregation and deformability in severe obese subjects. Int J Obes 2012; 36(3):342-7 DOI: 10.1038/ijo.2011.94]. Before Bariatric surgery, each patient included in the study gave a written informed consent to allow the bariatric medical team belonging to Department of Emergency and Organ Transplantation of the University of Bari “Aldo Moro” to use data of the study for research purposes only. The members of the bariatric medical team were formally accepted by the Director of the University Hospital of Bari.

## Sources of funding

Funds to Gabriella Garruti from the University of Bari “Aldo Moro” (Italian correspondent “Progetto di Ateneo”).

## Author contribution

Gabriella Garruti designed the study, was involved in the follow up of patients before and after surgery and wrote the paper.

Michele De Fazio was involved in the surgical procedures and in the discussion of results.

Palma Capuano was involved in patient selection and surgical procedures and in the discussion of results.

Gennaro Martinez was involved in the surgical procedures.

Maria T. Rotelli was coordinating the measurements of Adiponectin and Resistin and all the other laboratory tests reported in the paper.

Francesco Puglisi designed the study, was involved in patient selection and surgical procedures and in the discussion of results.

Nicola Palasciano was involved in the surgical procedures and in the discussion of results.

Francesco Giorgino was involved in the discussion of results.

## Registration of research studies

1. Name of the registry: http://www.researchregistry.com
2. Unique Identifying number or registration ID: Researchregistry5052
3. Hyperlink to the registration (must be publicly accessible):

## Guarantor

Gabriella Garruti is fully responsible for the work and the conduct of the study, had access to the data, and controlled the decision to publish on the behalf of all the Authors involved in the study.

## Consent

Before Bariatric surgery each patient included in the study gave a written informed consent to allow the bariatric medical team belonging to Department of Emergency and Organ Transplantation of the University of Bari Aldo Moro to use data for research pourposes only.

## Provenance and peer review

Not commissioned, externally peer reviewed.

## Declaration of competing interest

Authors declare that they have no competing financial interests in relation to the work described.
